# Digital divide in information verification among young seniors: An analysis of literacies and sociodemographic factors

**DOI:** 10.1371/journal.pone.0359202

**Published:** 2026-09-24

**Authors:** Wenting Yu, Yanxinyue Liu, Cindy Sing Bik Ngai, Jihye Park

**Affiliations:** Department of Language Science and Technology, The Hong Kong Polytechnic University, Hung Hom, Kowloon, Hong Kong, China; University of Udine: Universita degli Studi di Udine, ITALY

## Abstract

In the post-truth era, characterized by rampant misinformation, seniors are particularly susceptible to its ramifications. This study endeavored to identify how sociodemographic factors and five literacy dimensions were associated with intentional information verification among young seniors and bridge the digital divide in verification. Based on the empirical data collected from 405 young seniors aged 55–74 in Hong Kong, the findings indicated that education was associated with both literacy levels and verification behaviors. Different literacy dimensions showed distinct associations with information verification: media literacy and digital literacy emerged as core correlates of both interpersonal and institutional verification, while information literacy showed an additional association with institutional verification. This research enriches the conceptual comprehension of the connection between literacy and information verification and provides feasible insights for developing targeted interventions to narrow the digital divide in seniors’ verification engagement and mitigate the effects of misinformation.

## 1. Introduction

The rapid expansion of digital media, combined with persuasive techniques and weak gatekeeping, has dramatically fueled the globally unchecked spread of misinformation, threatening personal security, public health, and even national stability [1–3]. Compared to other age groups, seniors show higher susceptibility to misinformation and may face greater difficulty responding to misleading information in digital environments, reflecting persistent inequalities in digital skills and information outcomes [4,5]. A cross-national study of 6,068 adults showed that older adults were less likely to identify or perform corrections of misinformation on social media across the United States, the United Kingdom, Canada, and France [6]. *NewsGuard* found that four in five over-65s in the UK worried about misinformation, including health, politics and society, and climate [2]. Together, these findings suggest that older adults’ vulnerability may involve difficulties in both recognizing and correcting misleading content across multiple information domains, thereby revealing a verification-related dimension of the digital divide. Importantly, such vulnerability may be particularly salient among young seniors in developed societies, who tend to have greater information needs, propelled by continued participation in the workforce, intensified political engagement, substantial wealth, and propensity to place credence in misinformation [7,8]. The repercussions of misinformation-related vulnerability can be severe as they approach or surpass retirement age, given their relatively limited opportunities to recover financial losses and reduced resilience to emotionally manipulative tactics [9]. This may deepen the digital divide in verification capacity and exacerbate the challenges young seniors face in fully integrating into the information society in developed regions [10].

As a corrective remedy, encouraging information verification is important for mitigating the harmful effects of misinformation among young seniors [10]. Verification is conceptualized as a deliberate act aimed at evaluating and affirming authenticity [11]. In the present study, information verification is conceptualized as a general behavior that may occur across different information domains. Researchers have contended that prompting individuals to verify messages before trusting or disseminating them is an instrumental countermeasure to misinformation [12,13]. Despite frequent portrayals of seniors as primary targets of misinformation by political actors, foreign propagandists, and financial fraudsters, most survey-based and intervention-oriented studies rely on younger participants [11], leaving limited empirical research on seniors’ verification practices. Beyond examining verification behavior patterns, scholarship should also address interventions to foster verification, such as literacy interventions. Vraga et al. [14] stood among the earliest to explicitly articulate the conceptual nexus between news literacy and information verification. Prior research has also introduced various types of literacies and explored their respective impacts on verification. Nevertheless, scarce studies fully compare how different literacies affect information verification. In addition, understanding seniors’ heightened susceptibility to misinformation necessitates a thorough investigation into their sociodemographic characteristics. Past studies have also revealed that these attributes, such as socioeconomic status (SES) and social capital, influence people’s ability or intention [15–17]. However, limited attention has been given to how young seniors’ sociodemographic attributes relate to their literacy levels and verification intentions. Taken together, these gaps point to a verification-related digital divide, whereby inequalities in seniors’ socioeconomic resources and literacy may shape not only access to digital information but also the capacity to evaluate and verify it.

To address these gaps, we surveyed young seniors aged 55–74 in Hong Kong to assess their sociodemographic attributes, literacy levels, and information verification behaviors. Following a local Hong Kong policy report, this age group is referred to as “young-olds” [7]. Rather than being defined by a single statutory retirement age, it spans the transition from later working life to retirement and the early stage of older adulthood. We conducted a comparative analysis to investigate how different literacy dimensions (media, information, news, social media, and digital) were associated with two information verification types (interpersonal and institutional), and whether sociodemographic attributes were linked to literacy and verification behaviors. Because the measures did not refer specifically to any certain type of information, the findings primarily concern general interpersonal and institutional verification. By linking sociodemographic resources, literacy capacities, and verification outcomes, the study extends the digital-divide perspective beyond digital access and use to verification divide. Although the empirical focus is on young seniors in Hong Kong, the conceptual framework and findings may also be applied to other aging societies worldwide. The findings also inform the design of future interventions and policies to enhance seniors’ literacy.

## 2. Literature review and hypotheses development

### 2.1. Information verification and digital divide

Information verification refers to the structured process through which individuals assess the truthfulness, reliability, and authenticity of obtained information [11,12]. By integrating new information with pre-existing knowledge, people can identify inconsistencies and mitigate the uncertainty caused by such discrepancies through verification activities [12,17]. Engaging in information verification substantially reduces the risks associated with misinformation; however, it requires additional cognitive effort and digital competence. The willingness and ability to verify are therefore unevenly distributed among populations, reflecting the digital divide in verification [4,9,18].

The digital divide describes inequalities in individuals’ skills to access to, use of, and benefits gained from digital technologies such as the internet and smart devices [4,5]. It not only refers to differences in material access but also includes disparities in digital literacy, confidence, and adaptability to online environments. Seniors are often labeled as “digital refugees,” a term highlighting their struggle to adapt to the fast-evolving digital ecosystem [4]. They are particularly vulnerable to online misinformation due to age-related cognitive decline and lower digital literacy skills relative to younger adults [19]. Their diminished ability to discern falsehoods on digital platforms often leads them to inadvertently spread digital rumors and fall victim to online scams, jeopardizing both their personal and financial well-being [5]. Within this context, the effectiveness of information verification as a defense mechanism against misinformation is inherently constrained by the digital divide. Individuals with higher digital literacy are more capable of engaging in deliberate evaluative processes, while those with limited competence may not be able to do so effectively [19]. Information verification can therefore be understood as a digital outcome associated with unequal capacities to convert technological access into effective verification practices, revealing a verification divide.

Drawing on Tandoc et al.[13], information verification can be delineated into two types: internal and external. When individuals first encounter new information, they often assess its credibility by drawing upon their prior knowledge and experiences—a process known as internal verification. If doubts about the information persist after this initial assessment, they then turn to external verification [13]. Throughout the process of external information verification, individuals must seek diverse sources and pursue supplementary insights to enrich their understanding of pertinent topics, thereby fostering more enlightened and well-informed judgments [12]. This process draws upon both formal, systematically structured sources within established information frameworks and informal, experiential inputs embedded in daily life [17,20]. Accordingly, external verification is categorized into interpersonal and institutional aspects. Interpersonal verification encompasses the solicitation of corroborative opinions from informal, personal networks, including acquaintances, family members, or other social contacts, and examining peer-generated content, such as comments and online discussions. Conversely, institutional verification involves consulting authoritative, structured sources, including official organizations, search engines, and specialized fact-checking platforms (e.g., Google Search services and independent fact-checking websites) [13].

These two forms of external verification may occur either incidentally or intentionally. Incidental external verification denotes a passive reliance on others’ verification. In contrast, intentional external verification requires a more strategic and proactive approach, compelling individuals to actively seek validation through interpersonal or institutional sources [12,13]. Intentional verification, in contrast to incidental verification, typically entails considerable cognitive and labor effort, requiring heightened information retrieval and authentication, and compelling individuals to mobilize additional knowledge and reasoning to ensure information accuracy and credibility [12,13].

Specifically, this study focused on the actions involving the intentional verification of information through external sources, rather than passively relying on subsequently encountered corrective information or using one’s preexisting knowledge to discern misinformation. We believe intentional external verification, comprising interpersonal verification and institutional verification, is more effective in protecting people from being misled by false information for several reasons. Firstly, corrections are not always issued for false information, and even when they are, they may not reach the same audience as the original misinformation. The illusory truth effect posits that people have a tendency to perceive information as true upon first exposure, and repeated falsehoods can reinforce this mistaken belief [21]. Secondly, based on the priming effect, exposure to false information can generate “activation tags” that cognitively link related concepts [22]. These associations become more cognitively accessible and influence subsequent judgments by remaining salient or “top of mind” [23]. In this way, once misinformation is cognitively activated, it is likely to be retrieved and used when individuals assess later information. Thirdly, intentional external verification represents a modifiable behavior that individuals can develop through targeted interventions and active learning [3], while internal verification processes are less observable and harder to intervene in. Therefore, it is important to study the factors affecting intentional external verification in the misinformation age, because engagement in such verification may reflect whether individuals can convert access to digital information into active, protective responses to questionable content.

### 2.2. Associations between literacy and young seniors’ information verification

In digitally saturated and mediatized societies, literacy has become a crucial lifelong skill for seniors to bridge the digital divide, particularly in the context of population aging, longer life expectancy, extended working lives, delayed retirement, and inadequate pensions [8,24]. In 2014, 40% of persons aged 55 + were employed, and the U.S. Bureau of Labor Statistics forecasts indicated that, through 2024, the most rapid labor growth would occur in the 65–75 + age groups [25]. The Pew Research Center reported that American older workforce nearly quadrupled since the mid-1980s, reaching about 11 million by 2023, with 19% employment among those aged 65+ [8]. Parallel demographic-economic trajectories were evident internationally. As stated by *Euronews*, substantial proportions of Europeans remained economically active beyond ages 65 and 75 [26]. The Hong Kong Federation of Youth Groups has promoted workforce participation among the “young-olds” to mitigate the economic pressures associated with population aging [7]. Senior workers are engaging more actively with information consumption for various occupational purposes, yet encounter systemic hurdles including relatively lower media and information literacy [24], which heightens their vulnerability to online misinformation and deceptive content [9].

Within this context, literacy can be conceived as the individual capabilities, knowledge, and skills that constitute resilience against misinformation [27,28]. Based on this definition, a growing body of research emphasizes the broad potential of literacy as a tool for decreasing susceptibility to misinformation. Vraga et al. [14] contended that information verification is the behavior of news literacy (commonly regarded as a branch of media literacy) and serves as an epistemic tool for countering misinformation. However, different forms of literacy may provide distinct cognitive and practical resources for verification [19,29–31]. Previous research, for example, found that information literacy influenced the identification of fake news, whereas other forms of literacy did not demonstrate equivalent effects [29]. Accordingly, this study examines whether different literacy dimensions are differentially associated with the two types of information verification among young seniors.

***Media literacy*** denotes one’s competence in accessing, interpreting, scrutinizing, assessing, and generating messages across both traditional print and emerging digital media settings [32]. It enables individuals with a deeper understanding of the information production process, bolstering their awareness of the potential societal impacts of information, while simultaneously furnishing them with the practical capabilities essential for distinguishing between reliable and false information [3]. ***News literacy*** is notionally conceived as the intellectual foundation and capacities that permit individuals to grasp the personal and societal forces situated behind the processes of news creation, distribution, and reception, as well as the skills necessary to exercise agency within these procedures [1,14]. It prioritizes the ability to validate news content and judge its trustworthiness and accuracy and demands an appreciation of the sociocultural and personal factors that drive news generation, transmission, consumption, and the skill to thoughtfully manage these interactions [14].

Definitions of media literacy and news literacy converge on the capacity to discern misinformation and evaluate informational accuracy, thereby situating both literacies as viable resilience-oriented interventions for information verification. However, against the backdrop of an ever-evolving global information landscape facilitated by social media and AI algorithmic systems, an exclusive emphasis on news literacy and media literacy has proven insufficient in combating misinformation. Other emerging literacies (social media, information, and digital literacies) present their relevance and essentiality in addressing misinformation [19,29,30].

***Social media literacy*** is the ability that enables individuals to engage with and evaluate content across social media applications, such as Facebook and WeChat [30,31]. It emphasizes three key dimensions: technical expertise, referring to an in-depth understanding of platform operations; social interaction skills, which include the capacity to navigate, interpret, and handle digital interactions proficiently; and informational vigilance, encapsulating the key capacity to assess, verify, and authenticate online information [33]. ***Information literacy*** refers to the ability to identify information needs, locate and retrieve relevant sources, evaluate their credibility and relevance, and organize and use the acquired information ethically and effectively [20,29,34]. This notion underlyingly revolves around the capacity to recognize and fulfill informational needs while assessing sources for accuracy, authority, and relevance, making individuals accurately detect misinformation and deceptive content [20,29].

Furthermore, to bridge the digital divide, being equipped with ***digital literacy*** for young seniors is a must. It means nurturing a sharp and analytical mindset capable of carefully evaluating digital information and fortifying personal data security within an intensely connected and algorithm-driven informational ecosystem [29,32]. Possessing strong digital literacy connotes that individuals excel at utilizing various technological tools, including search engines and various platforms [32]. Such individuals can adopt analytical methods to appraise the validity and reliability of information, carefully interrogating the underlying motives, intentions, and biases of information sources [19].

However, it remains unclear how different literacy dimensions are associated with interpersonal and institutional verification. Based on the literature, this article posed the ensuing hypotheses:

**H1:** Young seniors with higher levels of media literacy (a), information literacy (b), news literacy (c), social media literacy (d), and digital literacy (e) are more likely to engage in interpersonal verification.
**H2:** Young seniors with higher levels of media literacy (a), information literacy (b), news literacy (c), social media literacy (d), and digital literacy (e) are more likely to engage in institutional verification.

### 2.3. Associations between sociodemographic attributes and young seniors’ literacy

The digital divide among older adults is deeply intertwined with sociodemographic factors shaping their ability to navigate and verify information in the digital environments [4,17]. Variables such as SES and social capital influence not only access to technological resources but also confidence and motivation for digital learning [35,36]. Seniors with higher SES or richer social networks receive greater emotional, material, and technical support, enhancing self-efficacy in adopting technologies and protecting against misinformation and online fraud [15–17]. However, aging-related physical and cognitive decline, limited media literacy, and technological anxiety often impede older adults’ digital participation [4,5]. This “grey digital divide” thus emerges not merely from technical barriers but from the cumulative effects of social background, resource inequality, and psychological resistance [4,37]. Such sociodemographic and aging-related disparities ultimately constrain seniors’ information verification capacity, intensifying vulnerability to digital misinformation. Additionally, given mixed findings on gender effects in literacy and misinformation exposure, we also included gender as a variable in our analysis.

Gender is frequently treated as a key demographic variable in literacy research, often linked to familiarity with and confidence in using digital technologies, which are historically regarded as male-oriented fields [38]. However, studies on gender disparities in literacy levels have revealed contradictory results. Some studies report a female advantage [39], others a male advantage [40], while some find no noticeable gender disparities in literacy levels [38]. These divergent outcomes raise the question:

**RQ1:** How is gender related to young seniors’ media literacy (a), information literacy (b), news literacy (c), social media literacy (d), and digital literacy (e)?

SES is also a key variable to assess literacy. Conceptually, SES represents individuals’ resource breadth and is typically operationalized by education and income in media effects research, which are two key determinants of digital divide [16]. Literacy variation is linked to educational attainment, partly due to more frequent and proficient use of digital and media devices for professional tasks and information gathering [38]. Higher education and income facilitate better access to digital technology and stronger critical thinking [41]. As articulated in the knowledge gap research, higher-SES individuals have greater exposure to digital technologies and higher proficiency with emerging information platforms [16,35]. Therefore, we proposed:

**H3:** Young seniors who are more educated have a higher level of media literacy (a), information literacy (b), news literacy (c), social media literacy (d), and digital literacy (e).
**H4:** Young seniors who possess higher income have a higher level of media literacy (a), information literacy (b), news literacy (c), social media literacy (d), and digital literacy (e).

We also considered the influence of social capital, which is broadly conceived as resources accessible through individuals’ social networks [36,42,43]. It comprises resources embedded in social ties with acquaintances, neighbors, friends, and family members [36,42,43]. These resources span social, affective, economic, and cultural domains and serve both instrumental (e.g., literacy guidance) and expressive functions (e.g., learning confidence), which are helpful for bridging the intergenerational digital divide [36]. In this study, we emphasized young seniors’ familial ties. One key variable pertains to marital status, under the premise that marriage can expand social networks by introducing new connections, through in-laws and a spouse’s acquaintances [41,44]. From a life-course perspective, bereavement in late adulthood may dissolve key social ties and weaken social connections, affecting seniors more than younger individuals [17]. Another factor is intergenerational co-residence, whereby younger family members under age 35 can support seniors by motivating digital device adoption [37]. The young generation not only facilitates access to technological devices but also provide foundational digital training for seniors [37,44]. Thereby, we proposed another two research questions:

**RQ2:** Compared to their peers, do those young seniors who are married demonstrate higher levels of media literacy (a), information literacy (b), news literacy (c), social media literacy (d), digital literacy (e)?
**RQ3:** Do young seniors who live with a family member under the age of 35 demonstrate higher levels of media literacy (a), information literacy (b), news literacy (c), social media literacy (d), digital literacy (e)?

### 2.4. The associations between sociodemographic attributes and young seniors’ information verification

As with gender differences in literacy, evidence on gender differences in information verification remains inconsistent. Chen et al. [45] suggested that women may be more predisposed to disseminating deceptive or misleading content. Men’s more positive dispositions toward technology and greater news consumption may promote more frequent verification [40]. Conversely, Laato et al. [46] found that females were less likely than males to believe or accept fake news. Women’s heightened concern about misinformation and stronger online social networks may facilitate information assessment and verification [17]. Hence, this article asked:

**RQ4:** How is gender related to young seniors’ interpersonal verification (a) and institutional verification (b)?

SES also exhibits its prominence in information verification. Knowledge gap theory posits that SES (education and income) is positively associated with knowledge acquisition [35]. It asserts that as information availability increases, knowledge disparities between individuals widen [35]. Increased media information primarily benefits higher-SES individuals, who have greater access and absorb information more quickly and effectively [16,35]. Prior studies show that lower-SES individuals are more vulnerable to misinformation (e.g., fake news, rumors, and conspiracy theories) due to weaker detection skills, lower caution, and reduced critical thinking [15,47]. Thus, we assumed:

**H5:** Young seniors who are more educated are more likely to conduct interpersonal verification (a) and institutional verification (b).
**H6:** Young seniors who have higher income are more likely to conduct interpersonal verification (a) and institutional verification (b).

Social capital encompasses the aggregate of real or potential resources derived from individuals’ “durable network” relationships [36]. Social capital operates through interpersonal mechanisms such as obligations, expectations, trust, and information access [42], facilitating misinformation detection and more sophisticated information verification [17]. Spousal relationships and intergenerational cohabitation are key forms of social capital for seniors, providing support through information monitoring, psychological reinforcement, and emotional regulation. Married seniors have a trusted partner with whom they can discuss misleading or alarmist content. Younger cohabitants can impart fact-checking knowledge (e.g., search-based verification) and remind seniors to remain cautious of misinformation. However, little research has explicitly examined whether marital status and intergenerational cohabitation are associated with young seniors’ information verification. Thereby, this study raised the following two questions:

**RQ5:** Compared to their peers, are those young seniors who are married more likely to conduct interpersonal verification (a) and institutional verification (b)?
**RQ6:** Are young seniors who live with a family member under the age of 35 more likely to conduct interpersonal verification (a) and institutional verification (b)?

**Fig 1:**
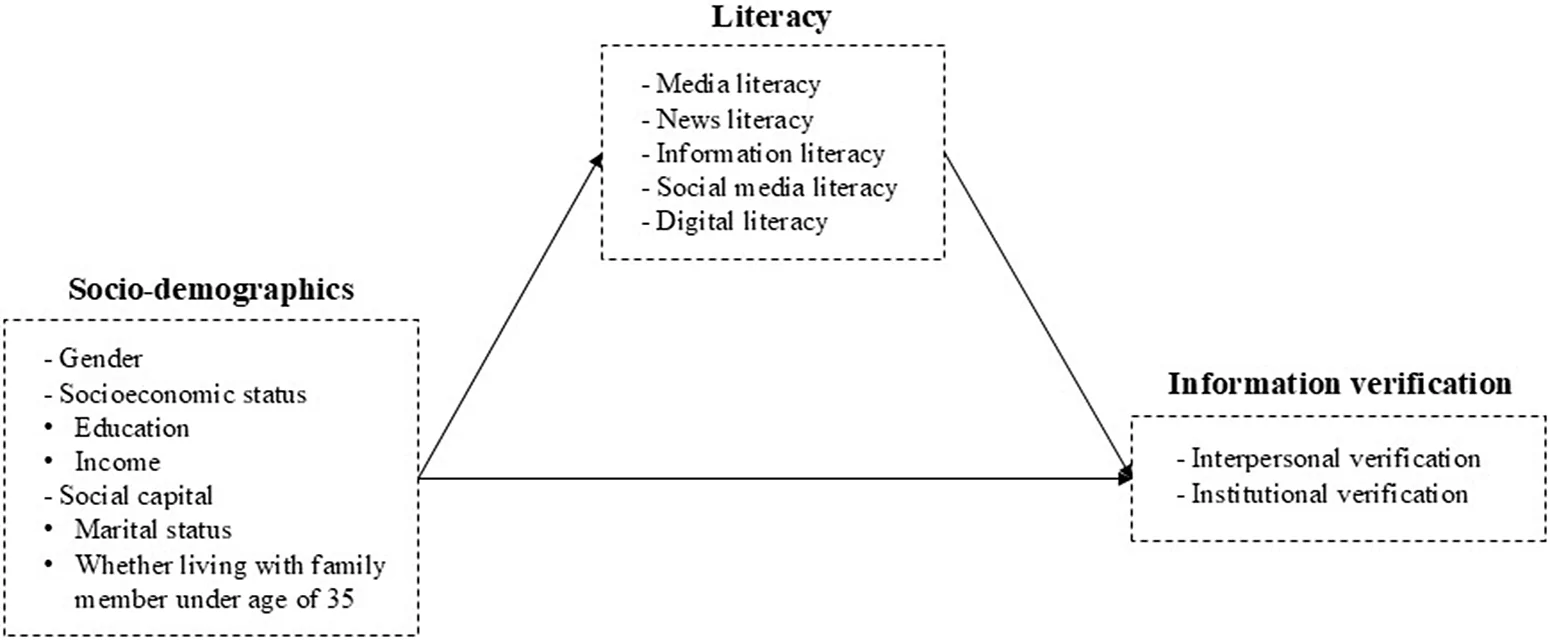
Conceptual model.

The conceptual model is shown in Fig 1. Assuming the proposed relationships are valid, it is possible that indirect relationships exist. Accordingly, we proposed the following research questions:

**RQ7:** Are the relationships between sociodemographic factors and interpersonal verification among young seniors statistically consistent with indirect associations through specific forms of literacy?
RQ8: Are the relationships between sociodemographic factors and institutional verification among young seniors statistically consistent with indirect associations through specific forms of literacy?

## 3. Method

### 3.1. Sample and procedure

This study aimed to identify factors related to young seniors’ proactive involvement in information verification. The study deliberately focused on adults aged 55–74 as a specific target population. This age range has been conceptualized in Hong Kong policy discussions as the “young-olds” stage [7]. Many people in this life stage remain engaged in employment, retirement planning, family responsibilities, and community activities, creating continuing needs to access, evaluate, and verify different forms of information [7]. Accordingly, age was used primarily to define a relatively bounded and policy-relevant population rather than as a focal explanatory variable. The analytical objective was to examine how socioeconomic resources and literacy-related capacities differentiated information verification within this predefined life stage, rather than to estimate a linear age gradient across the broader older population. Moreover, because the data were cross-sectional, any coefficient for chronological age would conflate within-group age variation with cohort-related differences in education, technological exposure, and media experience, and therefore could not be interpreted as an effect of aging. Age was therefore reported as a defining sample characteristic rather than included as a focal variable in the regression models.

Data collection was outsourced to a local survey company in Hong Kong and conducted from 25 September to 15 October 2024. The study received university ethical approval, and informed consent was obtained from all participants. Participants were recruited using gender quota sampling to obtain an approximately balanced distribution of male and female respondents. The final sample included 196 men (48.4%) and 209 women (51.6%). The relatively small proportion of final respondents with a bachelor’s degree or above, depicted in Table 1, may partly reflect cohort-specific educational opportunities, as many members of this age group completed their schooling before the substantial expansion of compulsory and higher education in Hong Kong [48].

**Table 1:** Descriptive demographic (N = 405).

Items	Frequency	Percentage (%)
*Gender*		
Male	196	48.4
Female	209	51.6
*Education*		
Primary and below	117	28.9
Junior secondary diploma	113	27.9
Higher diploma	123	30.4
Associate degree	11	2.7
Bachelor/professional diploma	39	9.6
Master or above	2	0.5
*Income*		
$0-4999	96	23.7
$5000-9999	63	15.6
$10000-19999	106	26.2
$20000-29999	82	20.2
$30000-39999	32	7.9
$40000-49999	15	3.7
$50,000 or above	11	2.7
*Marital Status*		
Single	12	3
Married	328	81
Divorced	29	7.2
Widowed	36	8.9
*Whether living with a family member under the age of 35 (excluding domestic servants)?*		
Yes	225	55.6
No	180	44.4

This survey comprised two parts. The first part collected demographics (gender, education, income, marital status, and co-residence with individuals under the age of 35, excluding domestic servants). The second part assessed five literacy dimensions and two information verification aspects. In total, 405 valid responses were retained for analysis, with demographics summarized in Table 1.

### 3.2. Measures

#### 3.2.1. Independent variables.

*Gender.* The survey used a binary gender classification. Males were coded 0 (48.4%) and females 1 (51.6%).

*Education.* Respondents reported highest education, which was coded from “1” to “6” from “primary and below” to “master or above” (*M* = 2.38, *SD* = 1.23).

*Income.* Participants estimated average monthly income, coded from “1” ($0–4999) to “7” ($50000 or above) (*M* = 2.95, *SD* = 1.55).

*Marital status.* Marital status was dummy-coded, with married participants serving as the reference category. Separate indicators were created for single, divorced, and widowed participants, with each variable coded 1 for membership in the respective category and 0 otherwise.

*Whether living with a family member under the age of 35 (excluding domestic servants).* This item assessed whether seniors lived with younger family members; responses were coded 0 = no (44.4.%) and 1 = yes (55.6%). Live-in domestic workers were excluded because they were treated as a distinct category of co-resident rather than as family members for this measure. Although they may provide household or caregiving assistance, digital information evaluation and verification are not among the duties specified in Hong Kong’s standard employment arrangements for domestic helpers [49]. Their capacity to provide such assistance also cannot be assumed, as employer–worker communication and language competence vary across linguistic backgrounds [50].

*Five types of literacy.* We measured all five literacy types on a 5-point Likert scale (1 = “strongly disagree,” 5 = “strongly agree” for four literacies; 1 = “very unfamiliar,” 5 = “very familiar” for literacy). These measures capture respondents’ self-reported or perceived literacy rather than objectively demonstrated knowledge or performance. Following Inan and Temur [51], media literacy was measured with four items, including “I use various media to follow news updates” (M = 3.26, SD = 0.65, Cronbach’s α = 0.65). As this Cronbach’s α fell within the 0.60–0.70 range, which is considered acceptable but not highly satisfactory [52], the findings involving media literacy were interpreted with caution. Drawing on Ullah and Ameen [34], information literacy included eight items, such as “I can effectively and efficiently obtain the information I need” and “I can identify which sources of information are relevant, authoritative, and reliable” (*M* = 3.50, *SD* = 0.64, Cronbach’s α = 0.92). Based on Ashley et al. [53], news literacy was measured with six items, including “Individuals can find news sources that reflect their political values,” and “Two people may view the same news report but receive different information” (*M* = 3.50, *SD* = 0.67, Cronbach’s α = 0.87). Social media literacy was measured with seven items from Puspitasari et al. [31], including “I can determine when to use social media as an information source” and “I know how to access various social media platforms and purposefully seek information” (*M* = 3.27, *SD* = 0.77, Cronbach’s α = 0.92). Following Hargittai and Hsieh [54], digital literacy was assessed with 12 familiarity items such as “How familiar are you with the advanced search functions of search engines (e.g., searching by time frame)?” and “How familiar are you with spyware (e.g., software installed on a phone or computer that collects and sells personal data or privacy information)?” (*M* = 2.70, *SD* = 0.91, Cronbach’s α = 0.96). We used the mean of each scale to indicate literacy levels across the five categories. Detailed measures are provided in S1 Appendix.

#### 3.2.2. Dependent variables.

*Information verification.* This study divided information verification into two types: interpersonal verification, and institutional verification [13]. The information verification measure reflects respondents’ self-reported verification behaviors, which are not directly observed. Adapted from Yu [55], respondents reported how often they engaged in specific actions to verify information they read, watched, or heard. A 5-point Likert scale was used (1 = “never,” 5 = “always”). Interpersonal verification was measured with five items such as “I ask family members or friends for their opinions and views” (*M* = 2.50, *SD* = 0.63, Cronbach’s α = 0.74). Institutional verification was measured using three items including “I check if mainstream news media (news websites, newspapers, TV, etc.) have published relevant information” (*M* = 2.56, *SD* = 0.80, Cronbach’s α = 0.74). Concrete scales are listed in S1 Appendix.

### 3.3. Analytic strategy

The study began with descriptive analysis using SPSS 24 to characterize the target respondents. The reliability of literacy and information verification measures was then tested. Before examining the hypothesized relationships among the variables, common method bias (CMB) was evaluated. Pearson’s correlation analysis was subsequently conducted to examine the bivariate relationships among the study variables, followed by the heterotrait–monotrait ratio of correlations (HTMT) to examine the discriminant validity. Then, multiple linear regression (MLR) analyses were conducted to test the hypotheses and address the research questions. After MLR, exploratory indirect-association analyses were conducted using PROCESS macro version 4.1. These analyses examined whether the observed associations between the study variables were statistically consistent with potential indirect relationships. Because all variables were measured concurrently using a cross-sectional design, the analyses were not intended to establish temporal ordering or causal mediation. Accordingly, the estimated indirect associations should be interpreted as exploratory rather than as evidence of an underlying causal mechanism [56].

## 4. Results

### 4.1. Common method bias (CMB)

To address potential measurement bias arising from the use of a single self-report survey, CMB was assessed using Harman’s single-factor test [57]. All measurement items were entered into an unrotated principal component analysis. According to the commonly adopted criterion, CMB may be considered a concern when one factor explains more than 50% of the total variance [58]. In the present study, the first factor explained 40.46% of the variance, which was below the recommended cutoff. Therefore, the findings suggest that CMB was unlikely to pose a serious threat to the study.

### 4.2. Correlations, discriminant validity and multicollinearity

Pearson’s correlation analysis was conducted (Table 2). Correlation coefficients with absolute values (|r|) closer to 1.0 were interpreted as indicating strong relationships, those with |r| between 0.3 and 0.7 as moderate, and those with |r| below 0.3 as weak [59]. Given the relatively high Pearson correlations among information, news, and social media literacy (*r*s = 0.70–0.73), we calculated HTMT to assess whether the five literacy dimensions were empirically distinguishable. Across the five literacy dimensions, the HTMT values ranged from 0.52 to 0.80 and were all below the conservative threshold of 0.85 [60]. These results suggest that the literacy dimensions were empirically distinguishable in this sample. Multicollinearity was examined using variance inflation factors (VIF). All VIF values (shown in Table 3), ranging from 1.10 to 3.02, were below 5.0, indicating no serious multicollinearity concerns [61].

**Table 2:** Pearson correlation coefficients for research variables.

Variables	M	SD	1	2	3	4	5	6	7	8	9	10	11	12	13	14
1. Gender	0.52	0.50	1													
2. Education	2.38	1.23	−.20**	1												
3. Income	2.95	1.55	−.24**	.76**	1											
4. Single	0.03	0.17	−.06	.17**	.14**	1										
5. Divorced	0.07	0.26	−.15**	.05	.00	−.05	1									
6. Widowed	0.09	0.29	.01	−.25**	−.28**	−.06	−0.09	1								
7. Whether living with a family member under the age of 35	0.56	0.50	.13**	.21**	.28**	−.17**	−.14**	−.26**	1							
8. Media literacy	3.26	0.65	−.04	.38**	.34**	−.01	−.01	−.13**	.20**	1						
9. Information literacy	3.50	0.64	−.10	.57**	.47**	.07	−.01	−.20**	.17**	.57**	1					
10. News literacy	3.50	0.67	−.07	.45**	.39**	.05	−.01	−.17**	.11*	.47**	.70**	1				
11. Social media literacy	3.27	0.77	−.13**	.53**	.47**	.08	.00	−.24**	.17**	.50**	.73**	.69**	1			
12. Digital literacy	2.70	0.91	−.16**	.61**	.52**	.07	−.09	−.24**	.23**	.42**	.55**	.48**	.63**	1		
13. Interpersonal verification	2.50	0.63	−.04	.31**	.30**	.01	−.04	−.10*	.15**	.43**	.38**	.32**	.41**	.55**	1	
14. Institutional verification	2.56	0.80	−.12*	.46**	.40**	.06	−.04	−.17**	.13**	.43**	.57**	.49**	.55**	.59**	.68**	1

Note: ** denotes that the correlation is significant at a confidence level of 0.01; * denotes that the correlation is significant at a confidence level of 0.05.

**Table 3:** Multiple linear regression analyses of information verification.

Variable	Interpersonal verification		Institutional verification		VIF
	*Model 1 Beta*	*Model 2 Beta*	*Model 3 Beta*	*Model 4 Beta*	
*Background factors*					
Gender	0.01	0.04	-0.03	-0.02	1.15
Education	0.20**	-0.15*	0.38***	0.02	3.02
Income	0.14	0.07	0.10	0.02	2.58
Single	-0.03	0.00	-0.03	-0.01	1.10
Divorced	-0.04	0.02	-0.07	-0.02	1.10
Widowed	0.00	0.05	-0.05	-0.01	1.18
Whether living with a family member under the age of 35	0.06	0.01	0.00	-0.03	1.29
*Literacy*					
Media literacy		0.24***		0.10*	1.57
Information literacy		0.02		0.21**	3.00
News literacy		-0.03		0.07	2.32
Social media literacy		0.05		0.08	2.91
Digital literacy		0.48***		0.33***	2.11
*Adjusted R²*	0.10	0.34	0.22	0.43	

Note: *Beta*, the standardized regression coefficient. ***p < 0.001, **p < 0.01, *p < 0.05. VIF = variance inflation factor.

### 4.3. MLR analysis

First, MLR analyses (Table 3) examined the associations between sociodemographic attributes and young seniors’ information verification (*H5-H6, RQ4-RQ6*). Education was significantly and positively linked with interpersonal (*β* = 0.20, *p* < 0.01, Model 1) and institutional verification (*β* = 0.38, *p* < 0.001, Model 3), corroborating H5(a, b). In contrast, income showed no correlation with either type of information verification behavior, leading to rejection of H6(a, b). Furthermore, gender (*RQ4*), marital status (*RQ5*), and living with a family member under the age of 35 (*RQ6*) were not related to information verification. Subsequently, after controlling for sociodemographic attributes, the analyses examined the associations between the literacy dimensions and information verification behaviors (*H1-H2*). In Model 2, higher levels of media literacy (*β* = 0.24, *p* < 0.001) and digital literacy (*β* = 0.48, *p* < 0.001) corresponded with more frequent interpersonal verification. In Model 4, media literacy (*β* = 0.10, *p* < 0.05), information literacy (*β* = 0.21, *p* < 0.01), and digital literacy (*β* = 0.33, *p* < 0.001) emerged as significant positive correlates of institutional verification. Hence, H1(a, e) and H2(a, b, e) were supported, whereas H1(b, c, d) and H2(c, d) were not.

A notable change in the association between education and interpersonal verification emerged after the literacy dimensions were introduced into the regression model. Education was positively associated with interpersonal verification in Model 1 (*β* = 0.20, *p* < 0.01); however, after the five literacy dimensions were added in Model 2, its coefficient became significantly negative (*β* = −0.15, *p* < 0.05). This sign reversal indicates that the education coefficient in the fully adjusted model represents its residual association with interpersonal verification after accounting for the variance shared with the literacy dimensions. The reversal also suggests that the relationship between education and interpersonal verification may be more complex than indicated by the initial positive coefficient and is examined further in the exploratory indirect-association analyses.

To examine the relationships between sociodemographic characteristics and young seniors’ literacy levels (*H3-H4, RQ1-RQ3*), the regression model (Table 4) substantiated that higher educational attainment corresponded with higher media literacy (*β* = 0.30), information literacy (*β* = 0.50), news literacy (*β* = 0.38), social media literacy (*β* = 0.41), and digital literacy (*β* = 0.52), with *p* < 0.001. Consequently, H3(a-e) were affirmed. Conversely, none of the five literacy dimensions varied significantly according to income, leading to rejection of H4(a-e). Table 4 also indicated that literacy scores showed no significant differences by gender (*RQ1*) or by co-residence with a family member under age 35 (*RQ3*). For marital status (*RQ2*), married young seniors served as the reference group. Compared with their married counterparts, single young seniors did not differ significantly across any of the five literacy dimensions. In contrast, marital dissolution showed some differences. Specifically, divorced young seniors scored lower in digital literacy (*β* = −0.13, *p* < 0.01), while widowed ones exhibited lower social media literacy (*β* = −0.10, *p* < 0.05) and digital literacy (*β* = −0.08, *p* < 0.05).

**Table 4:** Multiple linear regression analyses of literacy.

Variable	Media literacy	Information literacy	News literacy	Social media literacy	Digital literacy
	*Model 1 Beta*	*Model 2 Beta*	*Model 3 Beta*	*Model 4 Beta*	*Model 5 Beta*
*Background factors*					
Gender	0.03	0.02	0.03	-0.03	-0.06
Education	0.30***	0.50***	0.38***	0.41***	0.52***
Income	0.11	0.09	0.10	0.12	0.08
Single	-0.06	-0.03	-0.04	-0.02	-0.04
Divorced	-0.01	-0.03	-0.03	-0.04	-0.13**
Widowed	0.00	-0.05	-0.05	-0.10*	-0.08*
Whether living with a family member under the age of 35	0.10	0.02	-0.03	0.02	0.06
*Adjusted R^2^*	0.16	0.32	0.20	0.29	0.40

Note: *Beta*, the standardized regression coefficient. ***p < 0.001, **p < 0.01, *p < 0.05.

### 4.4. Exploratory indirect association analysis

*RQ7* and *RQ8* posited potential indirect associations among education, literacy, and the two forms of information verification behavior. The exploratory analyses were performed with the PROCESS macro [62], applying 5,000 bootstrap samples and 95% confidence interval. Significance was determined when its 95% bootstrap confidence interval did not include zero. Based on above analyses, media literacy and digital literacy were examined as potential intervening variables in the relationship between education and interpersonal verification (*RQ7*).

**Fig 2:**
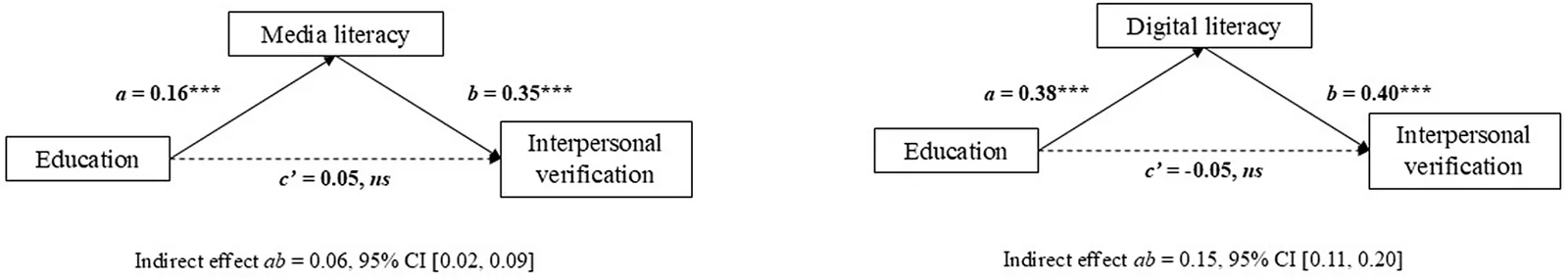
Exploratory indirect-association models linking education to interpersonal verification. Note. *p < 0.05, **p < 0.01, ***p < 0.001; *ns* = not significant. Solid arrows represent significant estimated associations, whereas dashed arrows represent nonsignificant estimated associations. Each literacy dimension was examined in a separate exploratory model.

The exploratory indirect-association analyses were conducted separately for media literacy and digital literacy, as demonstrated in Fig 2. The total association between education and interpersonal verification was positive and significant (*B* = 0.10, *Boot SE* = 0.04, 95% CI [0.03, 0.17]). Education was positively associated with media literacy (*B* = 0.16, *SE* = 0.04, *p* < 0.001), which was, in turn, positively associated with interpersonal verification (*B* = 0.35, *SE* = 0.05, *p* < 0.001). The indirect association through media literacy was significant (*B* = 0.06, *Boot SE* = 0.02, 95% CI [0.02, 0.09]), thereby confirming a potential indirect relationship between education and interpersonal verification through media literacy. The remaining direct association was positive but nonsignificant (*B* = 0.05, *Boot SE* = 0.04, 95% CI [−0.03, 0.12]). Education was also positively related to digital literacy (*B* = 0.38, *SE* = 0.04, *p* < 0.001), which was subsequently positively related to interpersonal verification (*B* = 0.40, *SE* = 0.04, *p* < 0.001). Notably, the indirect association was statistically significant (*B* = 0.15, *Boot* *SE* = 0.02, 95% CI [0.11, 0.20]), underscoring another potential indirect relationship through digital literacy. In contrast, the remaining direct association between education and interpersonal verification was negative but nonsignificant (*B* = −0.05, *Boot SE* = 0.04, 95% CI [−0.12, 0.02]).

Importantly, the decomposition of the education–interpersonal verification association differed across the two literacy models and helps clarify the sign reversal observed in Table 3. In the media literacy model, both the direct and indirect components were positive, indicating that media literacy represented part of the positive overall association but did not reverse the direction of the education coefficient. In contrast, the digital literacy model decomposed the positive total association into a larger positive indirect component through digital literacy and a negative residual direct component. The opposing signs are consistent with an inconsistent indirect-association pattern [63], although the negative direct component was not statistically significant. This pattern suggests that the initial positive association between education and interpersonal verification may partly reflect the higher digital literacy associated with education; once digital literacy was taken into account, the residual association of education shifted in a negative direction. Nevertheless, because Table 3 simultaneously included all five correlated literacy dimensions, the significant negative education coefficient cannot be attributed solely to digital literacy and may also reflect statistical suppression or the redistribution of shared variance.

For institutional verification (*RQ8*), MLR results also implied potential indirect associations through media literacy, information literacy and digital literacy. Across the three models, the total association between education and institutional verification was positive and significant (*B* = 0.25, *Boot SE* = 0.04, 95% CI [0.16, 0.33]). Education was positively associated with media literacy (*B* = 0.16, *SE* = 0.04, *p* < 0.001), while media literacy was positively associated with institutional verification (*B* = 0.37, *SE* = 0.06, *p* < 0.001). The bootstrapped indirect association was observed (*B* = 0.06, *Boot SE* = 0.02, 95% CI [0.02, 0.10]), indicating a pattern consistent with a potential indirect relationship. The remaining direct association was also positive and significant (*B* = 0.19, *Boot SE* = 0.04, 95% CI [0.11, 0.27]).

Moreover, another significant indirect relationship showed education was positively linked with information literacy (*B* = 0.26, *SE* = 0.03, *p* < 0.001), which in turn was positively correlated with institutional verification (*B* = 0.55, *SE* = 0.06, *p* < 0.001). The bootstrapped indirect-association estimate through information literacy was statistically different from zero (*B* = 0.14, *Boot SE* = 0.02, 95% CI [0.10, 0.19]), supporting its indirect role. A positive direct association between education and institutional verification remained statistically significant after information literacy was considered (*B* = 0.11, *Boot SE* = 0.04, 95% CI [0.02, 0.19]).

**Fig 3:**
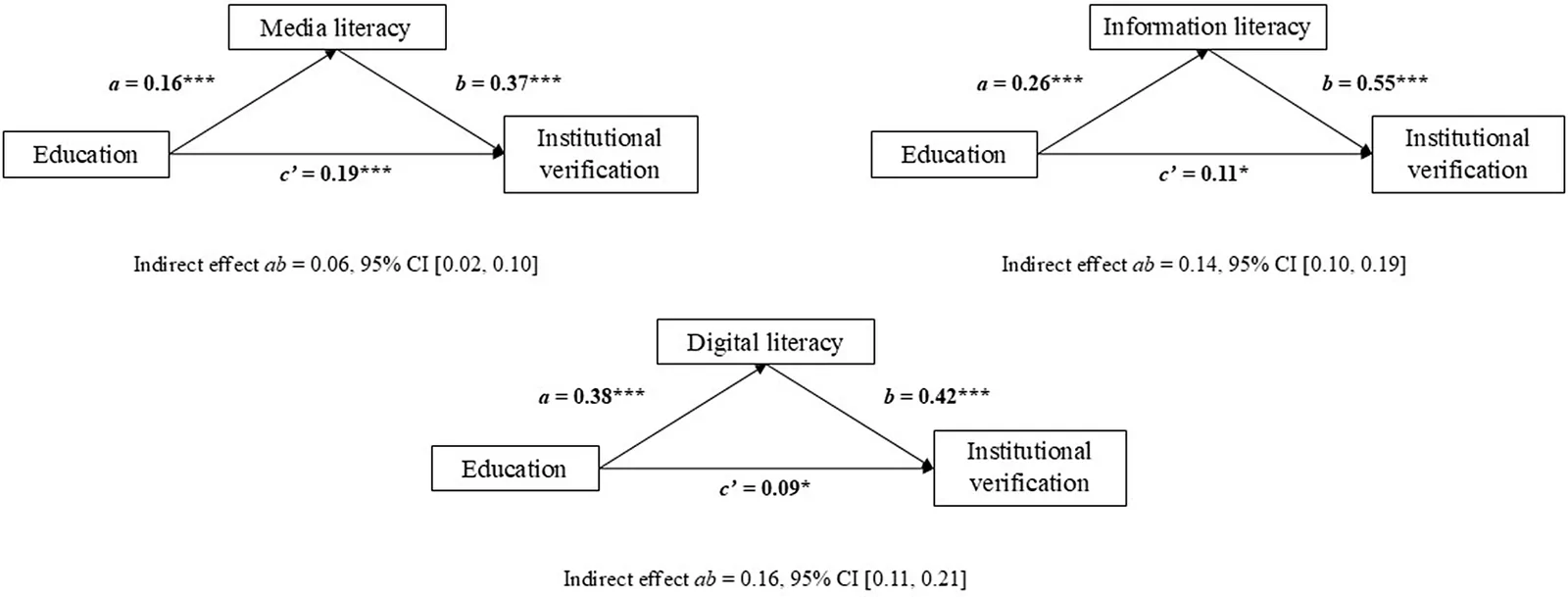
Exploratory indirect-association models linking education to institutional verification. Note. *p < 0.05, **p < 0.01, ***p < 0.001; *ns* = not significant. Solid arrows represent significant estimated associations, whereas dashed arrows represent nonsignificant estimated associations. Each literacy dimension was examined in a separate exploratory model.

Additionally, education was positively related to digital literacy (*B* = 0.38, *SE* = 0.04, *p* < 0.001), which was positively related to institutional verification (*B* = 0.42, *SE* = 0.05, *p* < 0.001). The bootstrapped estimate of the indirect association through digital literacy also differed significantly from zero (*B* = 0.16, *Boot SE* = 0.03, 95% CI [0.11, 0.21]), supporting the potential indirect role of digital literacy. Education also retained a positive and statistically significant direct association with institutional verification after digital literacy was taken into account (*B* = 0.09, *Boot SE* = 0.04, 95% CI [0.002, 0.17]). Fig 3 shows the indirect associations linking education to institutional verification.

Given the cross-sectional design, these findings should be interpreted as exploratory associations rather than evidence of causal mediation.

### 4.5. Robustness check

Given the strong correlation between education and income (*r* = 0.76), a robustness check was conducted to examine whether their estimated associations with literacy were sensitive to their simultaneous inclusion in the regression models (Table 5). Additional regressions were therefore estimated in which education and income were entered separately and then simultaneously, with the same background covariates included in all models. When entered separately, education was positively associated with all five literacy dimensions (*β*s = 0.37–0.57, all *p*s < 0.001), interpersonal verification (*β* = 0.29, *p* < 0.05), and institutional verification (*β* = 0.45, *p* < 0.05). Income was likewise positively associated with all five dimensions (*β*s = 0.32–0.46, all *p*s < 0.001), interpersonal verification (*β* = 0.28, *p* < 0.05), and institutional verification (*β* = 0.37, *p* < 0.05). In the joint models, education remained significantly associated with all five literacy dimensions (*β*s = 0.30–0.52, all *p*s < 0.001), interpersonal verification (*β* = 0.20, *p* < 0.01), and institutional verification (*β* = 0.38, *p* < 0.001). By contrast, the income coefficients decreased to 0.08–0.12 for the literacy dimensions and became nonsignificant. They were also attenuated for interpersonal verification (*β* = 0.14, *p* = 0.062) and institutional verification (*β* = 0.10, *p* = 0.178). This attenuation suggests that education and income shared substantial explanatory variance, with education showing the more consistent unique association when both variables were included.

**Table 5:** Robustness checks comparing the separate and joint entry of education and income across the five literacy dimensions and two types of information verification.

Outcome	*β*_*education*_ (separate model)	*β*_*education*_ (joint model)	*β*_*income*_ (separate model)	*β*_*income*_ (joint model)
Media literacy	0.37***	0.30***	0.32***	0.11, *ns*
Information literacy	0.56***	0.50***	0.45***	0.09, *ns*
News literacy	0.45***	0.38***	0.38***	0.10, *ns*
Social media literacy	0.49***	0.41***	0.42***	0.12, *ns*
Digital literacy	0.57***	0.52***	0.46***	0.08, *ns*
Interpersonal verification	0.29*	0.20**	0.28*	0.14, ns
Institutional verification	0.45*	0.38***	0.37*	0.10, ns

Given the moderate-to-strong correlations among the five literacy dimensions, additional robustness analyses were conducted to examine whether their estimated associations with verification were sensitive to their simultaneous inclusion in the regression models. Each literacy dimension was first entered separately after the same background covariates and was then compared with the original models in which all five literacy dimensions were entered simultaneously (Table 6). For interpersonal verification, all five literacy dimensions were positively associated with the outcome when entered separately, with standardized coefficients ranging from 0.22 to 0.57 (all *p*s < 0.001). Each dimension also explained significant additional variance beyond the background factors, with ΔR^2^ values ranging from 0.04 to 0.19. When the five dimensions were entered simultaneously, only media literacy (*β* = 0.24, *p* < 0.001) and digital literacy (*β* = 0.48, *p* < 0.001) retained significant unique associations. The coefficients for information, news, and social media literacy were substantially attenuated and became nonsignificant.

**Table 6:** Robustness checks comparing separate and simultaneous entry of literacy dimensions.

Literacy dimension	Interpersonal verification: separate model *β* (ΔR²)	Interpersonal verification: simultaneous model *β*	Institutional verification: separate model *β* (ΔR²)	Institutional verification: simultaneous model *β*
Media literacy	0.36*** (0.11***)	0.24***	0.30*** (0.07***)	0.10*
Information literacy	0.29*** (0.06***)	0.02, *ns*	0.44*** (0.13***)	0.21**
News literacy	0.22*** (0.04***)	−0.03, *ns*	0.34*** (0.09***)	0.07, *ns*
Social media literacy	0.34*** (0.08***)	0.05, *ns*	0.41*** (0.12***)	0.08, *ns*
Digital literacy	0.57*** (0.19***)	0.48***	0.48*** (0.13***)	0.33***

Note. Separate models included the same background covariates and one literacy dimension at a time. Simultaneous models included all five literacy dimensions together with the background covariates. Values in parentheses are changes in explained variance (ΔR^2^) after adding the respective literacy dimension. *p < 0.05, **p < 0.01, ***p < 0.001; *ns* = not significant.

For institutional verification, all five literacy dimensions were also positively associated with the outcome when entered separately (*β*s = 0.30–0.48, all *p*s < 0.001), accounting for significant increments in explained variance (ΔR^2^ = 0.07–0.13). In the simultaneous model, media literacy (*β* = 0.10, *p* < 0.05), information literacy (*β* = 0.21, *p* < 0.01), and digital literacy (*β* = 0.33, *p* < 0.001) retained significant unique associations, whereas news and social media literacy did not. These patterns indicate that the literacy dimensions shared substantial explanatory variance. Accordingly, nonsignificant coefficients in the simultaneous models should be interpreted as limited unique associations after adjustment for the other literacy dimensions, rather than as evidence that the corresponding literacy dimensions were unrelated to verification.

Note. Separate models included either education or income, along with the same background covariates. Joint models included education and income simultaneously. *p < 0.05, **p < 0.01, ***p < 0.001; *ns* = not significant.

## 5. Discussion

The study introduces a comprehensive, multi-dimensional assessment of literacy and verification behaviors among Hong Kong young seniors aged 55–74. We also examined which sociodemographic attributes are related to literacy levels and information verification behaviors among young seniors. The cross-sectional results showed that education, media literacy, and digital literacy were key correlates of effective information verification, whereas similar associations were not observed for other variables.

### 5.1. Differential associations of education and income with literacy and verification

As anticipated, our findings underscored education’s pivotal role in linking young seniors’ literacy to information verification, reconciling with existing study [15,38,41,47]. However, income did not show comparable effects. The robustness analyses further indicated that both education and income were positively associated with all five literacy dimensions and two types of verification when examined separately, but the income coefficients were substantially attenuated and became nonsignificant after education was included, while education retained significant unique associations. This pattern indicates that income was not substantively irrelevant; rather, much of its association with literacy and verification was shared with education.

Digital divide theory traditionally emphasized income as a crucial factor influencing access to digital and information technologies, but declining internet-based device costs have somewhat reduced income’s role in digital usage outcomes [64]. Hong Kong has achieved a high level of basic digital access, with Internet access and smartphone ownership approaching saturation at the population level. Hong Kong evidence reveals that widespread access and device ownership do not necessarily translate into digital knowledge, sophisticated use, or the ability to benefit from online resources, particularly among older adults [65,66]. Therefore, high Internet penetration and widespread digital infrastructure have diminished income’s significance as a determinant of literacy and information verification. Instead, critical thinking and the ability to learn and adapt to digital tools have become more influential. The knowledge gap hypothesis also posits that education is “a powerful correlate” of acquiring public affairs and scientific knowledge from mass media, because higher education is associated with stronger literacy and comprehension skills [35]. Our result suggested that digital inequalities among Hong Kong young seniors may increasingly concern skills and beneficial outcomes rather than basic technological access alone in digital divide. That is to say, widespread connectivity does not ensure that individuals possess the evaluative, informational, and technological capacities required to verify questionable content. Education-related resources, including comprehension, critical evaluation, and adaptability, may therefore show their significance once a basic level of access has been achieved.

The relationship between education and interpersonal verification appears to be more complex than a uniformly positive association. Education was positively associated with interpersonal verification before the literacy dimensions were included, but the coefficient became negative after all five dimensions were entered; similarly, the exploratory analysis showed that the positive total association comprised a positive indirect association through digital literacy and a negative, although nonsignificant, residual direct association. One possible explanation is that education facilitates interpersonal verification primarily by strengthening literacy-related capacities, especially the ability to access and evaluate information digitally. Once these capacities are held constant, however, more highly educated young seniors may be more accustomed to seeking and evaluating information independently. This interpretation is consistent with evidence that education is associated with more active information seeking among older adults and that participation in later-life learning may enhance self-confidence and self-dependency [67]. They may consequently place greater weight on formal or professional sources, which are generally perceived as highly credible by middle-aged and older adults in Hong Kong, and rely less on family members or friends for verification [68]. Statistically, the reversal may also represent a suppression-like pattern in which controlling for correlated literacy dimensions separates the positive literacy-related component of education from its remaining association with interpersonal verification [63,69]. Therefore, the negative coefficient should not be interpreted as evidence that education discourages verification. Rather, it may reflect differences in the literacy-related capacities and preferred routes through which verification is conducted. This pattern also illustrates how educational inequality may be associated not only with the likelihood of verification but also with the particular routes used to verify information. More highly educated young seniors may possess greater flexibility in moving between interpersonal discussion, independent online searching, and consultation of institutional sources.

### 5.2. Associations of social capital with literacy and information verification

This investigation also examined whether social capital factors (marital status and co-residence with a family member under the age of 35) were associated with literacy and information verification. Overall, neither factor showed consistent associations with the five literacy dimensions or the two forms of verification. This finding suggests that the presence of other household family members does not necessarily ensure informational or digital support. Co-residence alone does not capture the frequency, quality, or content of communication, nor whether younger family members actively assist with digital technologies or information evaluation. From an intergenerational digital-divide perspective, the nonsignificant associations of co-residence may point to a co-residence–support gap: the structural availability of younger family members within the household does not necessarily translate into activated digital or informational support.

Previous research has shown that younger family members may act as “warm experts [70],” engage in proxy Internet use [71], or provide digital back-feeding [72], but such support depends on their availability, willingness, and actual interaction with older family members rather than physical proximity alone. In contemporary urban societies, particularly in high-density and high-pressure environments such as Hong Kong, young adults often face occupational stress, long working hours, and time scarcity. These constraints may limit intergenerational interactions and reduce cognitive and informational support for seniors. Co-residence may therefore represent a latent support resource rather than realized assistance. This finding adds a relational dimension to the digital divide by suggesting that verification divide may also arise from differences in the capacity to mobilize household support for digital learning and check misleading information.

Additionally, marital status may be an imprecise indicator of the support that is actually available to young seniors. However, a nuanced difference emerged: marital dissolution was associated with lower literacy. Specifically, divorced seniors exhibited reduced digital literacy, whereas widowed individuals demonstrated diminished digital and social media literacy. These findings align with cognitive reserve theory, which argues that people with similar neuropathology can show markedly different late-life cognitive capacities (e.g., literacy level and verification intention) [73]. Such disparities may be attributed to lifelong influences that build cognitive reserve, including education, intellectually demanding occupations, and lifestyles (e.g., physical activity, and social interactions) [73]. In contrast, social network variables (e.g., marital status or residential situation) may have only limited or indirect associations with cognitive reserve, mainly through emotional support. Their associations were weaker than those of long-term educational investment and may partly reflect differences in individuals’ information needs. Nevertheless, unlike other literacy dimensions, digital and social media literacy are relatively novel abilities that require active learning and social interaction amid rapidly evolving technologies. For seniors, these new skills cannot be derived solely from prior knowledge or accumulated experience. As such, social isolation (e.g., marital dissolution or living alone) may hinder their ability to acquire and adapt to new digital tools effectively.

### 5.3. Associations of different literacy dimensions with information verification

This study found that media and digital literacy were significantly correlated with interpersonal verification, while media, information, and digital literacy were positively associated with institutional verification among young seniors. These findings underscored the particular importance of media and digital literacy for young seniors’ verification behaviors, suggesting that access to digital media does not necessarily ensure an equal capacity to respond to misinformation. While prior research has respectively examined five literacy forms in combating misinformation, deception, and rumor diffusion across various contexts [3,19,29,30,74], the present study specifically aimed to compare their relative impacts on intentionally external information verification. The results indicated that media and digital literacy were the consistent correlates of verification intention among young seniors, whereas other literacies were not comparably significant. Interestingly, the participants reported higher average levels of information literacy (*M* = 3.50), news literacy (*M* = 3.50), and social media literacy (*M* = 3.27) compared to media literacy (*M* = 3.26) and digital literacy (*M* = 2.70). This contrast indicates that lower descriptive mean scores did not preclude media and digital literacy from retaining statistically significant unique associations with both forms of information verification in the simultaneous models.

The robustness analyses provide a more qualified interpretation of the relationships between literacy and information verification. When examined separately, all five literacy dimensions were positively associated with both verification types. However, several coefficients were attenuated in the simultaneous models, indicating substantial shared variance among the dimensions. Digital literacy retained a statistically significant unique association with both forms of verification, possibly because verification increasingly requires users to navigate digital platforms, search for additional information, and access alternative sources [13,32]. These activities involve not only judging whether a claim appears credible but also knowing how to leave the original platform, formulate search terms, compare results, follow links, and locate alternative sources. Digital literacy may therefore function as a cross-cutting capacity that enables young seniors to translate verification intentions into concrete information-seeking actions. Media literacy also retained significant unique associations with both verification types, although its comparatively low scale reliability warrants caution in interpreting this finding. Media literacy may support verification by helping young seniors recognize persuasive techniques, distinguish evidence from opinion, and identify when a message requires additional checking [3,29].

Information literacy retained an additional unique association with institutional verification, which may reflect its particular relevance to locating, evaluating, and consulting formal or authoritative information sources. Institutional verification requires more than general skepticism: individuals must identify which organization possesses relevant expertise, locate its information, and determine whether the source is authoritative and applicable to the claim [13]. The nonsignificant coefficients for news and social media literacy in the simultaneous models should not be interpreted as evidence that these dimensions were unimportant. Rather, their significant associations in the separate models suggest that their contributions overlapped substantially with the broader evaluative and technological competencies captured by media, information, and digital literacy. The findings therefore support a differentiated account of literacy: several dimensions are relevant to verification overall, but digital, media, and information literacy explain comparatively distinct variance once their overlap is considered.

This research also examined potential indirect mechanisms. Findings revealed that media and digital literacy accounted for significant indirect associations linking education and interpersonal verification, while media, information, and digital literacy accounted for separate indirect associations between education and institutional verification. These patterns are consistent with the possibility that educational inequalities are reproduced in digital environments through unequal literacy-related capacities. More highly educated young seniors may be better positioned to acquire the evaluative and technological skills required to verify questionable information.

Taken together, these findings extend the digital-divide perspective beyond inequalities in access and use to a divide in information verification skills. Among young seniors, the critical divide may lie not simply in whether they are connected, but in whether technological access can be translated into the evaluative, navigational, and source-mobilization capacities required to challenge questionable information. From this perspective, information verification divide can be understood as the ability to transform digital access and literacy into protective epistemic action. Digital literacy and media literacy appear to provide a cross-cutting foundation for this conversion, whereas information literacy may channel it toward institutional verification.

### 5.4. Theoretical and practical contributions

To enhance digital inclusion, reduce vulnerability to scams and various misinformation, and promote informed civic participation among older adults, this study identified key factors that shield seniors from misinformation. Conclusively, this research has some theoretical implications. First, while existing study has proposed news literacy as an important indicator of verification [14], our comparison of five literacies showed that their impacts on verification were not uniform. Media literacy and digital literacy were associated with both verification routes, and information literacy showed an additional association with institutional verification. Comparing multiple literacy types advances theoretical understanding of the literacy–verification relationship. We argue for a more nuanced account of each literacy type’s distinct role, inviting theoretical refinement and differentiated measurement in future studies.

Second, our findings reveal that education, rather than income or other sociodemographic factors, is the primary correlate of young seniors’ literacy and information verification behaviors. This underscores the unique association of education with seniors’ digital and informational resilience, and that digital inequality in a highly connected society may be structured less by basic access alone and more by unequal cognitive, evaluative, and learning resources. The findings therefore extend digital-divide research beyond device ownership and digital access toward inequalities in verification skills, identifying information verification divide as a form of digital divide.

This study offered several practical implications. Since the study assessed general information verification, these implications primarily apply across information contexts. First, because digital literacy showed the strongest association with both interpersonal and institutional verification, it should be treated as a foundational intervention priority. Training should focus on the concrete digital actions required for verification, including formulating effective search terms, opening and comparing multiple search results, tracing claims to their original sources, checking publication dates, and navigating government or professional websites. Rather than simply instructing participants to “check official information,” training should teach them to determine which institution has relevant authority or expertise, verify the organization’s name and website domain, examine its stated responsibilities and contact information, distinguish official accounts from similarly named or imitative pages, and cross-check a claim across more than one authoritative source. These skills could be developed through step-by-step simulation tasks based on realistic social media posts, news headlines, or fraudulent messages. For example, participants could be asked to locate the original source of a claim, compare it with at least two independent sources, identify inconsistencies across platforms, and explain why a particular source should or should not be trusted. Repeated guided practice, followed by gradually more independent exercises, would help young seniors translate digital knowledge into actual verification behavior.

Second, the association between media literacy and verification indicates a need to strengthen young seniors’ ability to recognize when information requires further checking. Community workshops could use realistic misinformation examples to teach participants to identify warning signs such as unclear authorship, missing or unverifiable evidence, emotionally provocative wording, exaggerated certainty, misleading headlines, selective presentation of facts, and images presented without sufficient context. Participants could be guided to ask a set of structured questions: Who produced this message? What is the central claim? What evidence is provided? Is the evidence traceable? What information may have been omitted? Could the wording or visual presentation be designed to provoke an immediate reaction? Small-group discussion or role-play could then be used to help participants explain their judgments. Because the study found that co-residence with younger family members was not itself associated with stronger literacy or verification, practical programs should not assume that physical proximity automatically produces digital support. Instead, they should create structured opportunities that convert potentially available intergenerational relationships into active informational assistance, thereby helping to narrow the intergenerational digital divide.

Third, the consistent associations involving education suggest that standardized programs may not benefit all participants equally. Interventions should provide additional scaffolding for young seniors with less formal education, including plain-language instructions, repeated demonstrations, slower pacing, visual examples, and opportunities for assisted practice. Affordable lifelong-learning programs may be especially useful when they integrate basic digital navigation with critical information evaluation and verification exercises. Together, these differentiated interventions may help narrow a verification-related digital divide by enabling young seniors not only to access digital information but also to evaluate it and mobilize appropriate interpersonal or institutional resources.

### 5.5. Limitations and future directions

This study has several limitations. First, its cross-sectional design, exploratory indirect analyses, and reliance on self-reported measures preclude causal conclusions. The literacy measures may reflect perceived competence and confidence level rather than objective ability [75,76], while reported verification frequency may be affected by social desirability because verification is socially regarded as responsible behavior [77,78]. Future research should therefore employ longitudinal or experimental designs, performance-based literacy assessments, and observed verification behavior. Second, we examined individual-level sociodemographic factors (e.g., gender, education, income, co-residence) but not broader social factors, such as seniors’ external social networks (size, density, diversity, frequency, and proximity) in online and offline contexts, which may also be linked to literacy and information verification. Future studies should include these external sociological dimensions. Third, age was not modeled because adults aged 55–74 were treated as a predefined target population. Nevertheless, meaningful age-related differences may exist within this range and should be examined using more fine-grained age measures. Future studies should collect exact age and use age-adjusted and longitudinal designs to distinguish conditional age differences from cohort-related variation. Fourth, media and digital literacy retained statistically significant associations with both forms of information verification among young seniors. However, our findings may be most applicable to developed societies like Hong Kong, where devices and internet access are highly prevalent. Future research should test whether these patterns hold in regions with lower technological penetration. Finally, the study involved multiple statistical tests without formal adjustment for multiple comparisons, which may have increased the risk of Type I error. The findings should therefore be interpreted cautiously and replicated in future research.

## Supporting information

S1 Appendix Supplementary materials related to the measures reported in this study. (DOCX)

S1 Data Dataset underlying the findings reported in this study. (SAV)
