# Supplementary material for: Digital divide in information verification among young seniors: An analysis of literacies and sociodemographic factors
Source: PLoS One. 2026 Sep 24;21(9):e0359202. doi: 10.1371/journal.pone.0359202 (PMC13606963; doi:10.1371/journal.pone.0359202)
Supplement: Supplementary file 1 [file pone.0359202.s001.docx]

**Appendix 1: Measurements of Literacy and Information Verification**

**Literacy**

*Media literacy.*

(1) I use various media to follow news updates.

(2) I contact news organizations to express my reactions and criticisms.

(3) I discuss the news I see with family or friends.

(4) I remind those around me to be aware of the negative effects of the media.

*Information literacy.*

(1) I can effectively and efficiently obtain the information I need.

(2) I can identify which sources of information are relevant, authoritative, and reliable.

(3) I can recognize my information needs.

(4) I can assess the relevance and quality of information sources.

(5) I can use information in a legal and ethical manner.

(6) I can organize collected or generated information in a logical manner.

(7) I can effectively use selected information to complete specific tasks.

(8) I can critically evaluate the authenticity of information.

*News literacy*.

(1) The owners of media companies influence the content of the media.

(2) Individuals can find news sources that reflect their political values.

(3) Two people may view the same news report but receive different information.

(4) News coverage of political election candidates can influence people’s opinions.

(5) News is often more dramatic than the actual situation.

(6) Reports about conflicts are more likely to attract attention.

*Social media literacy.*

(1) Conflicting information and viewpoints on social media make me feel confused when learning about a topic.

(2) I can determine when to use social media as an information source.

(3) I know how to access various social media platforms and purposefully seek information.

(4) I prefer to obtain information from social media because posts on social media usually reflect my views.

(5) I know how to cite information obtained from social media platforms.

(6) I know how to initiate a discussion on social media.

(7) I know techniques that help in finding useful information on social media.

*Digital literacy.*

(1) How familiar are you with the advanced search functions of search engines (e.g., searching by time frame)?

(2) How familiar are you with document editing (e.g., entering and editing text)?

(3) How familiar are you with spyware (e.g., software installed on a phone or computer that collects and sells personal data or privacy information)?

(4) How familiar are you with Wikipedia?

(5) How familiar are you with clearing your web browsing history?

(6) How familiar are you with phishing websites?

(7) How familiar are you with tagging others on social media (e.g., tagging topics or friends in a Facebook post)?

(8) How familiar are you with photo editing (e.g., using photo manipulation tools)?

(9) How familiar are you with online forums (e.g., posting in an online forum)?

(10) How familiar are you with malicious software (e.g., Trojan viruses)?

(11) How familiar are you with scanning QR codes?

(12) How familiar are you with mobile payments (e.g., electronic payments such as ApplePay, Alipay, or digital Octopus)?

**Information verification.**

*Interpersonal verification*

(1) I ask family members or friends for their opinions and views.

(2) I ask experts in the relevant field whom I know for their opinions and views.

(3) I check the opinions and views of the general public regarding this information.

(4) I ask people I know for their opinions and views.

(5) I share and ask for opinions on online forums or social media.

*Institutional verification*

(1) I use search engines to find more related information.

(2) I check if mainstream news media (news websites, newspapers, TV, etc.) have published relevant information.

(3) I check if fact-checking websites or accounts have published relevant information.
